# Impact of an elastic sphere with an elastic half space revisited: Numerical analysis based on the method of dimensionality reduction

**DOI:** 10.1038/srep08479

**Published:** 2015-02-16

**Authors:** I. A. Lyashenko, V. L. Popov

**Affiliations:** 1 Sumy State University, 40007 Sumy, Ukraine; 2 Berlin University of Technology, 10623 Berlin, Germany; 3 National Research Tomsk Polytechnic University, 634050 Tomsk, Russia

## Abstract

An impact of an elastic sphere with an elastic half space under no-slip conditions (infinitely large coefficient of friction) is studied numerically using the method of dimensionality reduction. It is shown that the rebound velocity and angular velocity, written as proper dimensionless variables, are determined by a function of only the ratio of tangential and normal stiffness ("Mindlin-ratio"). The obtained numerical results can be approximated by a simple analytical expression.

Impacts of solid particles are of interest for many physical and technological processes related to the dynamics of granular media^1^,^2^,^3^,^4^. Even if the particles have a spherical shape and the material is purely elastic, the detailed dynamics of the impact can be very complicated and include partial slip, gross slip or no slip in the contact area during different phases of the impact. This is the reason why a comprehensive theory of frictional impacts has not been developed so far. The exact analytical solution exists only for the simplest case when complete sliding (gross slip) occurs in the whole contact area during the entire impact. However, already for the other limiting case of *no slip* in the whole contact area (infinitely large coefficient of friction) the complete solution has not been obtained so far in spite of the apparent simplicity of the problem. The classical theory using only the conservation laws and the rolling conditions, which can be found in textbooks on mechanics^5^, is intrinsically inconsistent as it considers the impact as being elastic but at the same time uses the condition of rigid rotation of the body as a whole. The tangential compliance of the contact as well as micro slip in the contact area has been taken into account first by Maw, Barber and Fawcett^6^ (MBF theory), based on the theory of normal contact for elastic spheres by Hertz^7^ and the theory by Mindlin for tangential contacts^8^. Barber later provided an analytical theory for only those phases of the impact during which complete adhesion takes place^9^. The MBF theory was validated experimentally by the authors themselves^10^ as well by other authors^11^,^12^,^13^,^14^. A review of existing impact models and their validation can be found in the book^15^. In spite of the long history of studies of impacts, the existing results still cover only part of the theoretically possible impact parameters, and no effective numerical methods for analyzing impact processes could be developed so far.

In the present paper we consider one of the simplest cases of an impact: an impact with no-slip in the contact area during the whole time of contact. Theoretical foundation for the solution of this problem was created by Hertz^6^ and Mindlin^8^ and is used in the MBF theory. The reason why this problem still has not been studied exhaustively is the mathematical complexity of the combined, time dependent normal and tangential problem. In a series of recent papers, Popov et. al. have shown that the theory by Hertz-Mindlin can be reproduced exactly by a contact of properly modified profile shape with a linear elastic foundation consisting of independent springs^16^,^17^,^18^,^19^,^20^. The method can be used not only for the combined normal-tangential contact with arbitrary history of loading but also for the rolling contact^21^. This method, called method of dimensionality reduction, MDR, simplifies the contact problem drastically and opens new ways for analytical and numerical treatment of dynamic normal and tangential contacts.

The paper is organized as follows. We first reproduce, for comparison with known exact results, the classical solution based on the assumption of rigid rotation at the last moment of contact. We then solve a simplified model with a constant contact stiffness, which provides the general understanding of the problem and the dimensionless variables which are of interest and will be used in the following analysis. After this the impact problem is solved using the method of dimensionality reduction.

## Results

### Simplified model of the impact with no-slip condition

#### Classical “rigid body” solution

**Figure 1:**
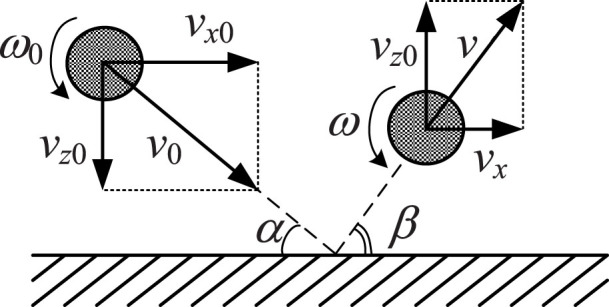
Schematic presentation of an impact.

Let us consider an impact of an elastic sphere with mass *m* and radius *R* on an elastic half space, as shown in Fig. 1. Let the moduli of elasticity of the sphere and the half space be *E*_1_ and *E*_2_, their Poison's numbers *ν*_1_ and *ν*_2_, and their shear moduli *G*_1_ and *G*_2_, accordingly. The main notations are illustrated in Fig. 1: The incident velocity of the center of mass of the sphere is *ν*_0_ with horizontal and vertical components *ν_x_*_0_ and *ν_z_*_0_, the incident angular velocity *ω*_0_, the rebound velocity is *ν* with components *ν_x_* and −*ν_z_*_0_, the grazing angle is *α*, and the rebound angle *β*.

We first reproduce the classical solution of the impact problem. Let *F_x_* and *F_z_* be the components of contact force acting on the sphere during the impact. The equations of motion of the sphere in the integral form can be written as
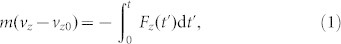

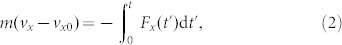

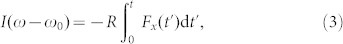
where *t* is the duration of the impact, and *I* = (2/5)*mR*^2^ is the moment of inertia of a homogeneous sphere. Together with the rolling condition for the tangential rebound velocity,
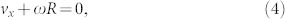
these equations determine unambiguously all kinematic quantities of the sphere after the impact:
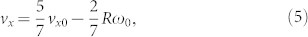

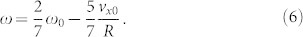


It can be easily seen that the impact is non-elastic, as the energy change during the impact,
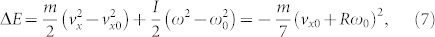
is negative. This solution is, however, oversimplified. While equations (1)–(3) are exact (under assumption of very short impact time), the kinematic condition (4) is intrinsically controversial: it cannot be valid during the whole time of impact, and its application to the last moment of impact is an arbitrary and not substantiated assumption. In reality, due to the elasticity of the sphere, the condition (4) will be valid *only* at one point in time during the impact.

#### Impact in the case of a constant contact stiffness

**Figure 2:**
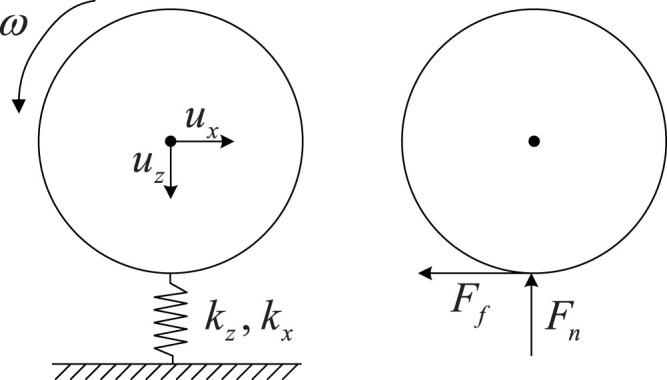
Simplified contact model with a constant contact stiffness (left) and the force diagram during the impact (right).

In a second step let us take into account the normal and tangential compliance of the contact in a simplified way. The normal and tangential compliance of the contact are changing during the impact due to changing contact configuration. Let us simplify this situation by considering an impact of a rigid sphere having a linear spring in the contact region. This also can be an elastic sphere with a flat patch. Due to the flat the contact stiffness will be constant provided the contact radius does not change considerably during the impact. The considered system and notation are shown in Fig. 2.

Equations of motion can be written as
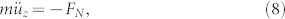

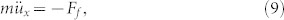

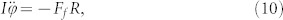
where
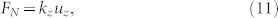

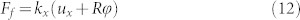
are the normal and tangential components of the contact force. In the last equation we took into account the fact that the tangential displacement of the contact point is a sum of the displacement of the center of mass and the displacement due to rigid rotation. The solution of the set of Eq. (8)–(12) with the initial conditions *u_x_*(0) = 0, 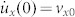, *u_z_*(0) = 0, 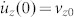, *ϕ*(0) = 0, 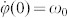 has the form
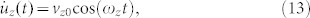

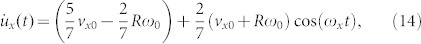

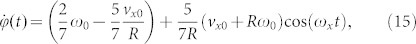
where 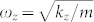, and 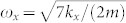. The duration of the impact, *t_i_*, is determined by the Eq. (13) and is equal to *t_i_* = *π*/*ω_z_*. The velocities at the last moment of the impact are equal to
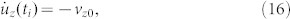

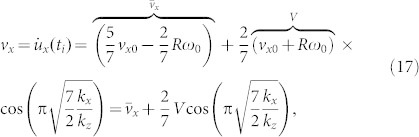

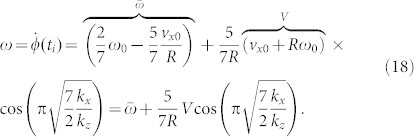
The energy change during the impact is equal to
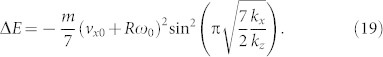
Note that the expressions for 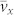 and 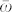 are exactly the classical solutions (5) and (6), while the remainder of Eq. (17) and (18) describes the influence of the finite tangential compliance. From Eq. (17) and (18), it follows that the combinations
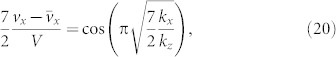

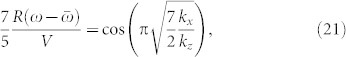

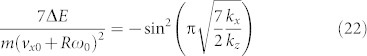
are functions of the ratio *k_x_*/*k_z_* of the normal and tangential contact stiffness. This suggests that the structure of the relations (20) and (21) may be valid for a more general case of contact of any shape. Indeed, for an arbitrary rotationally symmetric body the ratio of differential tangential and normal stiffnesses is constant and equal to Ref. 8 *k_x_*/*k_z_* *=* G*/E*, where
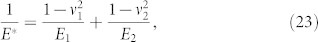

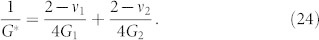


In Refs. 22 and 23 it was shown by numerical simulations that this is valid even for randomly rough fractal surfaces. We thus may anticipate that the Eq. (20) and (21) are valid for both regular forms and rough profiles, while the exact dependence may be replaced by another, shape dependent function. We arrive at the hypothesis that in the general case the relations (20) and (21) have to by replaced by
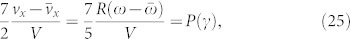

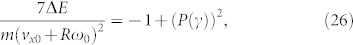
where
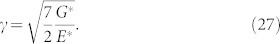
In the next Section, we will prove this hypothesis by numerical simulations and find the form of the function *P*(*γ*).

### Impact of a sphere: results of modeling

**Figure 3:**
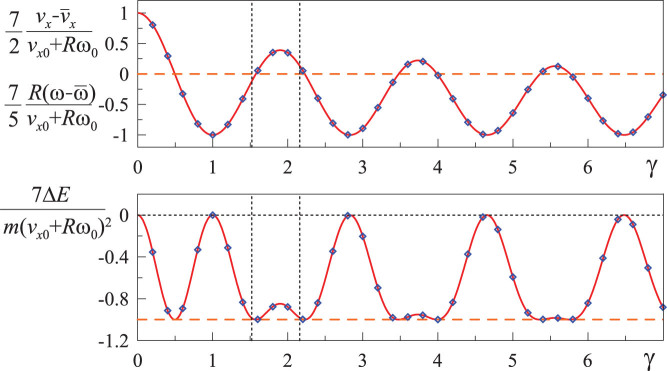
Dependencies of the variables 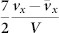, 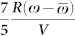, and 
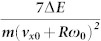
 on the parameter 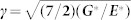. The impacts were calculated for various initial conditions, various radii, elastic moduli and masses of the spheres. Independently of the parameters used, all data collapse to a master curve, which determines unambiguously the rebound values of the kinematic variables as a function of the incident values. The classical, rigid-body solution (5), (6) is shown by the horizontal dashed line. The interval (28) is shown by vertical dotted lines.

In the present work, the equations of motion (8)–(10) were solved by the Euler integration procedure. The results for the energy change during the impact as a function of the parameter 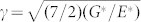 are presented in Fig. 3, where the dimensionless variables 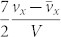, 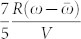, and 
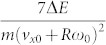
 are plotted as a function of the parameter *γ* defined by Eq. (27). Note that if the bodies have equal Poisson numbers: *ν*_1_ = *ν*_2_ = *ν*, then G*/E* = 2(1 − *ν*)/(2 − *ν*). From the thermodynamic stability criterion, it follows that Ref. 24 −1 < *ν* ≤ 1/2. Thus, for isotropic bodies, 2/3 < G*/E* < 4/3 which corresponds to
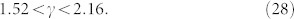
However, for anisotropic (e.g. orthotropic) media, the effective ratio G*/E* can be in a wider range than given by this Equation. We therefore present results outside the region (28) as well.

**Figure 4:**
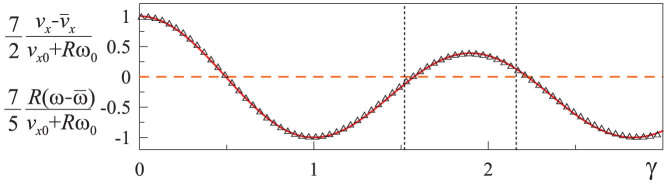
Dependencies of the variables 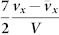, 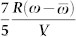 on the parameter 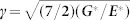 in the range of most interest for isotropic materials are shown with solid line. The range 1.52 < *γ* < 2.16 corresponding to Poisson number between −1 and 0.5 is marked by vertical dotted lines. The approximation (29) is shown with triangles; the classical, rigid-body solution (5), (6) is shown by the horizontal dashed line. The coefficient of determination for approximation (29) and numerical dependency in the full showed range of 0 < *γ* <3 is *R*^2^ > 0.9995.

Fig. 4 is a magnified representation of the most practically relevant range of the variable *γ*. In this range the numerically determined function *P*(*γ*) can be approximated with
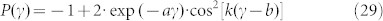
with *a* = 0.195, *b* = 0.061, and *k* = 1.685. For practically important case of *ν* = 1/3 we get *P* ≈ 0.20, and for incompressible media (*ν* = 1/2), *P* ≈ −0.09.

## Discussion

In the present paper, we used the method of dimensionality reduction in the area of its exact applicability (contact of axis-symmetric bodies) to simulate an impact of an elastic sphere on an elastic half-space. The main result of the study is the proof of the hypothesis (25) as well as numerical determination of the function *P*(*γ*) appearing in this equation. This function is presented in Fig. 3. A simple analytical approximation (29) was found for this function. We investigated a much wider range of the ratios G*/E* than would be relevant for isotropic elastic bodies. As for anisotropic bodies (e.g. media with orthotropic elasticity), this ratio can in principle have arbitrary values. The suggested method can be generalized straightforwardly to impacts of bodies of different form (not necessarily spherical), impact with adhesion, contacts with dry friction or impact of viscoelastic bodies.

## Methods

For simulation of normal and tangential contact during the impact we use the method of dimensionality reduction (MDR). In the framework of the MDR, two preliminary steps are performed^19^: First, the three-dimensional elastic bodies are replaced by a one-dimensional linearly elastic foundation consisting of an array of independent springs, with a sufficiently small separation distance Δ*x* and normal and tangential stiffness Δ*k_z_* = E*Δ*x* and Δ*k_x_* = G*Δ*x*. In the second step, the three-dimensional profile *z* = *f*(*r*) is transformed into a one-dimensional profile *g*(*x*) according to
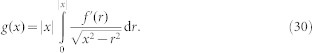
When the MDR-transformed profile *g*(*x*) is indented into the elastic foundation and is moved normally and tangentially according to an arbitrary law, the force-displacement relations of the equivalent one-dimensional system will reproduce those of the initial three-dimensional contact problem (proofs have been done in Refs. 18, 19.) The MDR solution has the same accuracy as the solutions by Cattaneo^25^ and Mindlin^8^: in the case of general Poisson ratio, there is an inaccuracy, which has been shown to be generally quite small^26^.

For a sphere with radius *R*, the shape in the vicinity of the contact is given by *f*(*r*) = *r*^2^/(2*R*). The one-dimensional MDR-image, according to (30), is *g*(*x*) = *x*^2^/*R*. If the vertical displacement *u_z_* of the center of mass is counted from the moment of first contact, it coincides with the indentation depth, and the vertical displacement of the spring of the elastic foundation at the point *x_i_*, 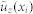, is equal to 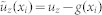 inside the contact region. The contact radius *a* is determined by the condition 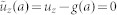. Due to the assumed stick condition in the whole contact region, the temporal incremental changes 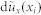 of the tangential displacements of all springs that are in contact with the profile, are equal to the rigid-body movement of the contact point, 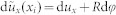. The normal and tangential forces are calculated easily as 
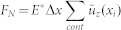
, 
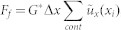
, where summation is over all springs in contact at the given moment of time.

## Author Contributions

V.L.P. and I.A.L. developed the model and the numerical algorithm, I.A.L. carried out computer simulations and prepared figures, V.L.P. wrote the main manuscript text. Both authors reviewed the manuscript.
